# Neonatal thermal response to childbirth: Vaginal delivery vs. caesarean section

**DOI:** 10.1371/journal.pone.0243453

**Published:** 2020-12-09

**Authors:** Anna Lubkowska, Sławomir Szymański, Monika Chudecka

**Affiliations:** 1 Chair and Department of Functional Diagnostics and Physical Medicine, Faculty of Health Sciences, Pomeranian Medical University, Szczecin, Poland; 2 Department of Obstetrics and Pathology of Pregnancy, Pomeranian Medical University in Szczecin, Szczecin, Poland; 3 Institute of Physical Culture Sciences, Faculty of Physical Education and Health, University of Szczecin, Szczecin, Poland; University of Mississippi Medical Center, UNITED STATES

## Abstract

Newborns, regardless of the method of termination of pregnancy, are exposed to the first exogenous stress factors during delivery. The purpose of the study was to evaluate the differences in newborns’ thermal response to vaginal (VD) vs caesarean section (CS) delivery. The temperature was measured during the first minutes of life within 122 healthy full-term newborns, on the forehead, chest and upper-back by infrared camera (FLIR T1030sc HD). The lowest temperatures were recorded in the forehead of VD newborns (significantly difference with CS; p < 0.001), the warmest was the chest. A significant correlation was found between the duration of the second stage of natural childbirth and surface temperature and pO2 in the newborn blood. The temperatures of selected body surface areas correlate highly positively, regardless of the mode of delivery. In the case of healthy neonates, with normal birth weight and full-term, VD creates more favourable conditions stimulating the mechanisms of adaptation for a newborn than CS.

## Introduction

Stress response is a fundamental requirement for the survival of the human species. All human beings, regardless of the method of termination of pregnancy, are exposed to the first exogenous stress factors during delivery. It is postulated that concluded that vaginal delivery (VD) is significantly more stressful in comparison to elective CS, as indicated from the higher serum cortisol levels observed in the VD [1]. Perinatal stress is strongly related to long-term programming of the hypothalamic–pituitary–adrenal (HPA) axis, regulates the body's adaptive processes and response to stimulating factors in the course of ontogenesis, influencing psychosomatic development [2]. For many years, comparative studies have been conducted on the effect of the mode of delivery on the early and distant consequences for children's psychosomatic traits. The increased risk of disease associated with immune function in the offspring [3–9], sensitisation allergens, and asthma and respiratory complications [10–15], metabolic and hormonal disorders [16–21] and the effect on colonisation of the gut flora correlated with gastrointestinal symptoms [22–25] has been repeatedly evaluated, and the results of these studies are consistent. However, no current reports regarding the comparison of the thermal response of newborns born by CS vs. VD, describing the scope and distribution of body temperature of newborns immediately after delivery have been found in the literature.

The temperature relationship between foetus and mother results from a combination of endogenous heat production by the foetus and the surrounding core temperature of the mother. The temperature different between the foetus and the mother is called the “heat clump” and is relatively constant [26]. The foetal temperature is approximately 0.2–0.5°C higher than the maternal temperature in utero and the foetus’ peripheral and core temperatures are almost exactly the same. The foetus is entirely dependent on the mother for temperature regulation and the uterus is a conduit for heat loss on the placenta from the foetus to the uterine wall (for approximately 85% heat loss), owing to a positive foetal-maternal temperature gradient [27–29]. The two most important inhibitors of non-shivering thermogenesis (NST)–being strong anti-lipolysis inhibitors–passed via the placenta to the foetus: adenosine and prostaglandin E2. Both of them play a very important role in: 1. the metabolic adaptation of a physiological hypoxic foetus (because NST requires adequate oxygenation); 2. their presence allows the foetus to accumulate an adequate amount of brown adipose tissue (BAT) before birth [26].

After birth, the child, by changing its environment, is exposed to lower temperature. The skin surface temperature is related to the difference between the body temperature and that of the environment. The temperature gradient between the infant and the air in a delivery room is usually around 10°C and creates the potential for heat loss to the air and poses a considerable threat to temperature homeostasis. The body temperature of infants drops directly after birth because of their disproportionate body mass-to-surface ratio, not fully developed control and executive mechanisms of thermoregulation, especially poor cutaneous vasoconstriction and vasodilation control, small amounts of subcutaneous fat and thin skin with increased permeability [30–33]. Such uncontrolled losses place the infant at risk for significant morbidities and mortality, therefor thermal homeostasis is essential for survival.

The major source of heat loss in the first weeks of life is evaporative heat loss that occurs when water is lost through the skin, undergoing conversion from a liquid to a gas, as during the delivery [34–36]. The skin of an infant is covered with amniotic fluid and usually vernix caseosa. Evaporation from the body surface of amniotic fluid and of water in amniotic fluid causes a loss of heat. At the same time, the infant is exposed to a colder temperature than it has experienced in utero. Exposure to colder environment may give rise to thermogenic responses that will increase basal heat production and the skin circulation may decrease to lower the heat losses [37]. The human body uses three responses to process the thermoregulatory information: afferent sensing, central regulation, and efferent autonomic responses which is initiated by a change in temperature by neurones that have thermo-sensitive receptors present in the skin, deep tissues, spinal cord and brain [38].

When an infant is born, in order to maintain its body’s core temperature, they go through discrete physiological and behavioural responses, initiated by the hypothalamus and cutaneous temperature receptors [39]. What constitutes a normal temperature in the newborn is still unclear, with a wide range of temperatures being accepted as normal. Previous research allowed to select important, thermally representative and, above all, suitable for a non-invasive thermographic analysis of the body surface areas of a healthy, term, vaginally delivered newborn and assess the range and distribution of temperatures within the forehead, back and chest areas [40]. The goal of our research was to estimate the potential effect of the mode of delivery (CS vs. VD) on: 1. the range of temperature changes in selected newborn’s body surface areas immediately after delivery; 2. the nature of the temperature distribution on its body; and 3. selected birth parameters of the newborn, which was achieved by including in the study and analysis a group of newborns born by C-section.

## Material and methods

The research was carried out from January 2018 to November 2019 in the Gynaecology and Obstetrics Ward, SPZOZ Hospital (an independent public healthcare institution) in Choszczno. The study included a total of 122 healthy newborns; 48 of them were delivered by caesarean section, while 74 vaginally delivered, full-term, healthy newborns were a comparative group. All of the vaginal deliveries included in the analysis took place without the use of instrumental assistance (obstetrical forceps or vacuum extractor).

The study was approved by the Bioethics Committee of the Pomeranian Medical University in Szczecin (KB 0012-15/12). The participants were informed about the course of the research and gave their written consent to participate. Immediately after birth, the temperature of selected body surface areas was recorded with an IR camera, without wiping the newborn, before further activities such as placing them in an open radiant warmer, and also before the setting of neonatal clinical care. The newborn was supported by the obstetrician under the arms in a vertical position and the temperature was measured on the anterior surface, in the chest region; on the posterior surface, in the upper-back region; and in the newborn’s forehead region. The analysis only included the thermograms of those newborns who scored 9–10 points on the Apgar scale (from the first minute), a medical qualification confirming the newborns’ condition and the healthy status of the newborns. For each selected body surface area, the mean surface temperature (T_mean_) was calculated. Thermal images were taken with a thermal imaging camera (FLIR T1030sc HD) in the delivery room, with all necessary precautions and without affecting the course of labour. The measuring procedures were carried out following the standards of the European Thermographic Association [41]. During imaging, the ambient temperature and humidity were constant at the measurement site, at appropriately 26°C and 55–60%. The camera was positioned in a straight line to the subject, 1.5 meters from the newborn's body. Skin emissivity was adopted as 0.98. The FLIR Tools software was used to analyse the thermograms. Additionally, data on maternal age, pregnancy order, as well as newborn sex, birth weight, body length, pregnancy week on delivery, and newborn blood gas test results (pH, pO2, HCO3, ctCO2, sO2) were collected.

### Statistical analysis

Statistical analyses were performed with the use of the STATISTICA 11 software (StatSoft, Poland). The values of the analysed parameters were normally distributed (which was verified with the Shapiro-Wilk test). We presented the results of the measurements as arithmetic means, standard deviations, and minimum and maximum values. To estimate the significance of differences in the temperature of selected body surface areas within the group of newborns born by CS and to compare the values of all analysed newborn traits depending on the mode of delivery the analysis of variance (ANOVA), post hoc Tukey’s test was used.

To estimate the relationship between T_means_ values of examinated body surface areas in the newborns from CS group the Pearson's correlation test were performed (r indicates correlation coefficient). The Spearman test was performed to verify the relationship between the mode of delivery and selected parameters of newborn blood gas tests (pH, pO2, HCO3, ctCO2, sO2) and the temperature of selected body surface areas. In addition, Spearman’s rank-order correlation coefficients between the duration of the second stage of labour and selected neonatal parameters were calculated.

## Results

Descriptive statistics of the selected parameters for mothers and newborns from both groups, i.e. those born by caesarean section and by vaginal delivery, are summarised in Table 1. The groups of women did not differ in terms of age and pregnancy order. There were also no statistically significant differences in anthropometric traits and newborn blood gas parameters. The duration of pregnancy in both groups was comparable, ranging between 37 and 41 weeks.

**Table 1:** Descriptive statistics of the values of maternal and neonatal traits under analysis.

	CS delivery				Vaginal delivery				
	Mean	Standard Deviation	Min	Max	Mean	Standard Deviation	Min	Max	Anova Post hoc Tukey
Maternal age [yrs]	28.14		5.84		17	41	27	5.66	18	41	0.497
pregnancy order	1.89		1.12		1	5	1.80	1.03	1	5	0.803
pregnancy week	38.94		0.89		37	40	38.5	2.59	37	41	0.474
birth weight [g]	3373		488		2300	4760	3430	523	2340	4960	0.72
body length cm]	55.11		3.15		48	62	55.7	3.38	47	64	0.544
pH	7.35		0.06		7.3	7.48	7.36	0.07	7.19	7.49	0.894
pCO2 [mmHg]	41.83		6.92		29.5	64.5	40.67	7.36	29.2	64.5	0.447
pO2 [mmHg]	30.17		7.22		17	43	30.03	6.83	17	44	0.897
HCO3 [mmol/l]	22.93	2.03	16.6	26.1	22.16	2.14	16.6	26.1	0.129
ctCO2 [mmol]	17.69	5.72	5	37.5	17.61	5.76	5	40.9	0.937
sO_2_%	51.75	17.77	17	79.6	51.17	16.72	17	82.5	0.996

In the conducted study, the temperature of selected body areas (forehead, chest and back) were measured in newborns born by CS and VD. The lowest temperatures were recorded in the forehead area of newborns, especially in those born by vaginal delivery. The range of these temperatures ranged from 33.2 to 37°C, while in newborns born by CS from 34.2 to 37.1°C. The warmest area in both cases was the chest, for which the thermal ranges for the groups are: 34.3 to 37.2°C (VD) and 34.3 to 37.3°C (CS), respectively.

When analysing in detail the temperature distribution within the examined group of newborns born by caesarean section, no statistically significant differences were found between the mean values of temperatures of selected body surface areas. Interestingly, the previous study of newborns from natural childbirth has shown such a variation in body area temperatures, indicating a statistically significantly lower temperature in the forehead area of newborns.

The temperatures of selected body surface areas between newborns from two groups differing in the mode of delivery were successively compared (Table 2). It was shown that the only area statistically significantly different in terms of temperature was the forehead, with the temperature values of this region of the body in newborns delivered vaginally being significantly lower compared to those delivered by caesarean section (difference 0.6°C).

**Table 2:** Descriptive statistics of the temperature values of selected body surface areas in newborns born by CS and VD.

	CS delivery				Vaginal delivery				
	Mean	Standard Deviation	Min	Max	Mean	Standard Deviation	Min	Max	Anova Post hoc Tukey
T_mean_ back (°C)	35.74	0.64	34.5	37.2	35.51	0.72	34	37	0.165
T_mean_ chest (°C)	35.86	0.71	34.3	37.3	35.64	0.68	34.3	37.2	0.162
T_mean_forehead (°C)	35.62	0.68	34.2	37.1	35.05^#^	0.74	33.2	37	0.00125**

** significance level of p < 0.01 –between groups.

# significance level of p < 0.05 –between T_mean forehead_ and both T_mean chest & back_ in vaginally delivered newborns.

**Fig 1:**
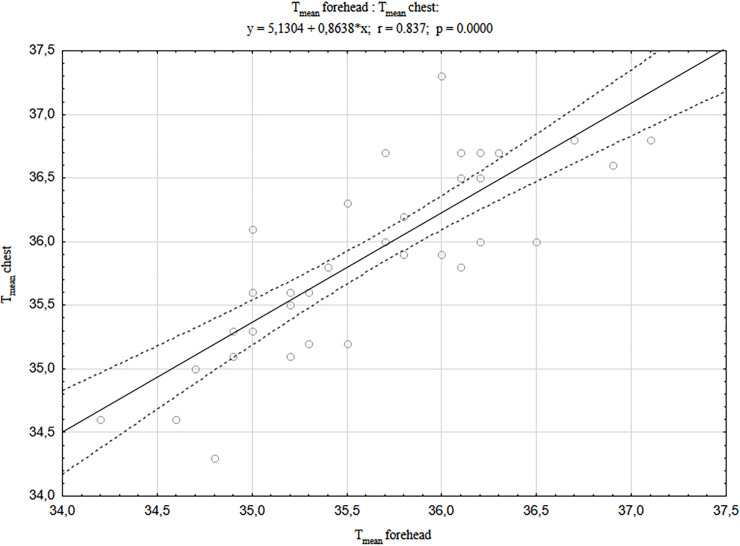
A correlation between T_mean_ forehead and T_mean_ chest in newborns born by CS.

**Fig 2:**
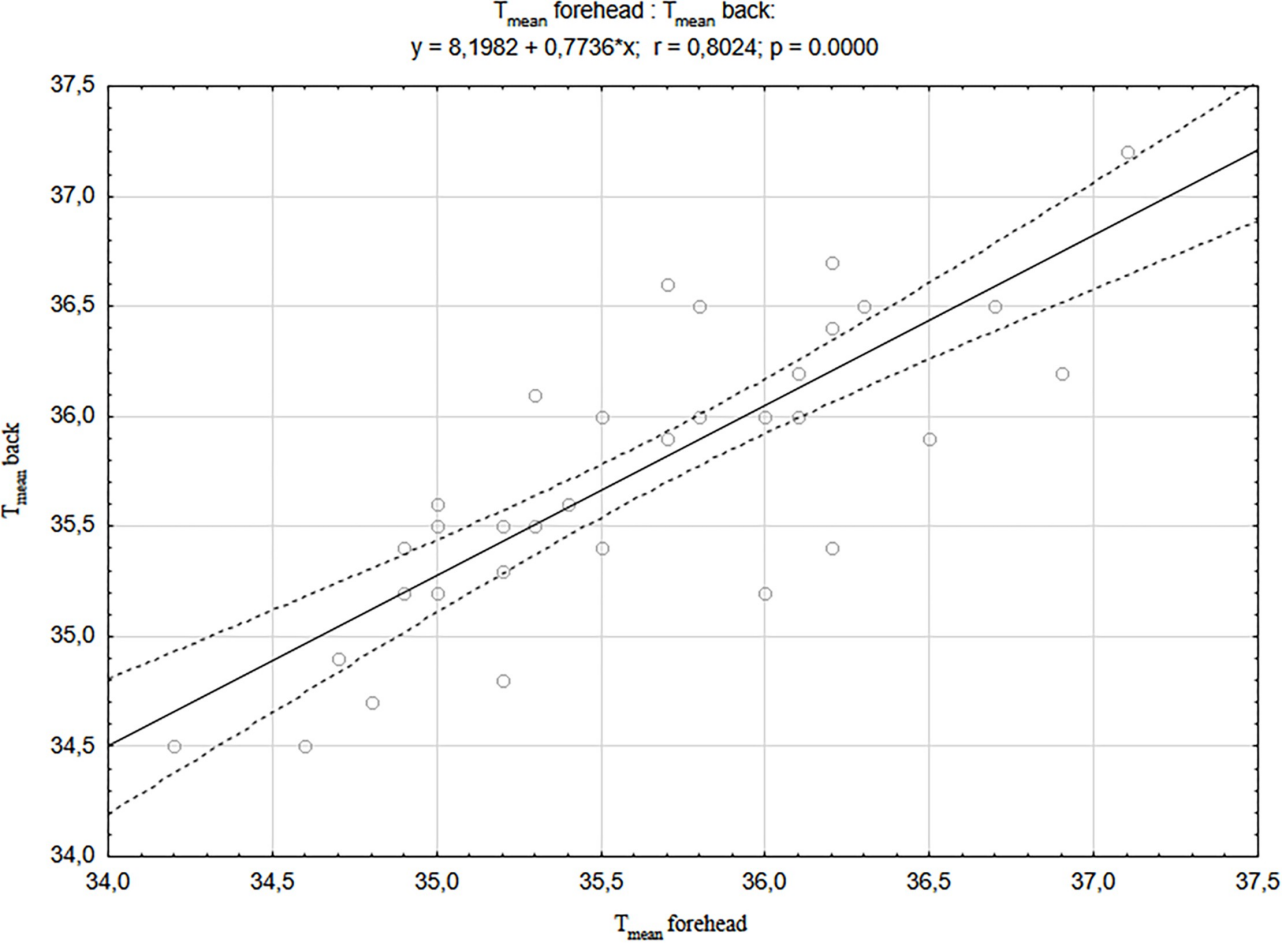
A correlation between T_mean_ forehead and T_mean_ back in newborns born by CS.

**Fig 3:**
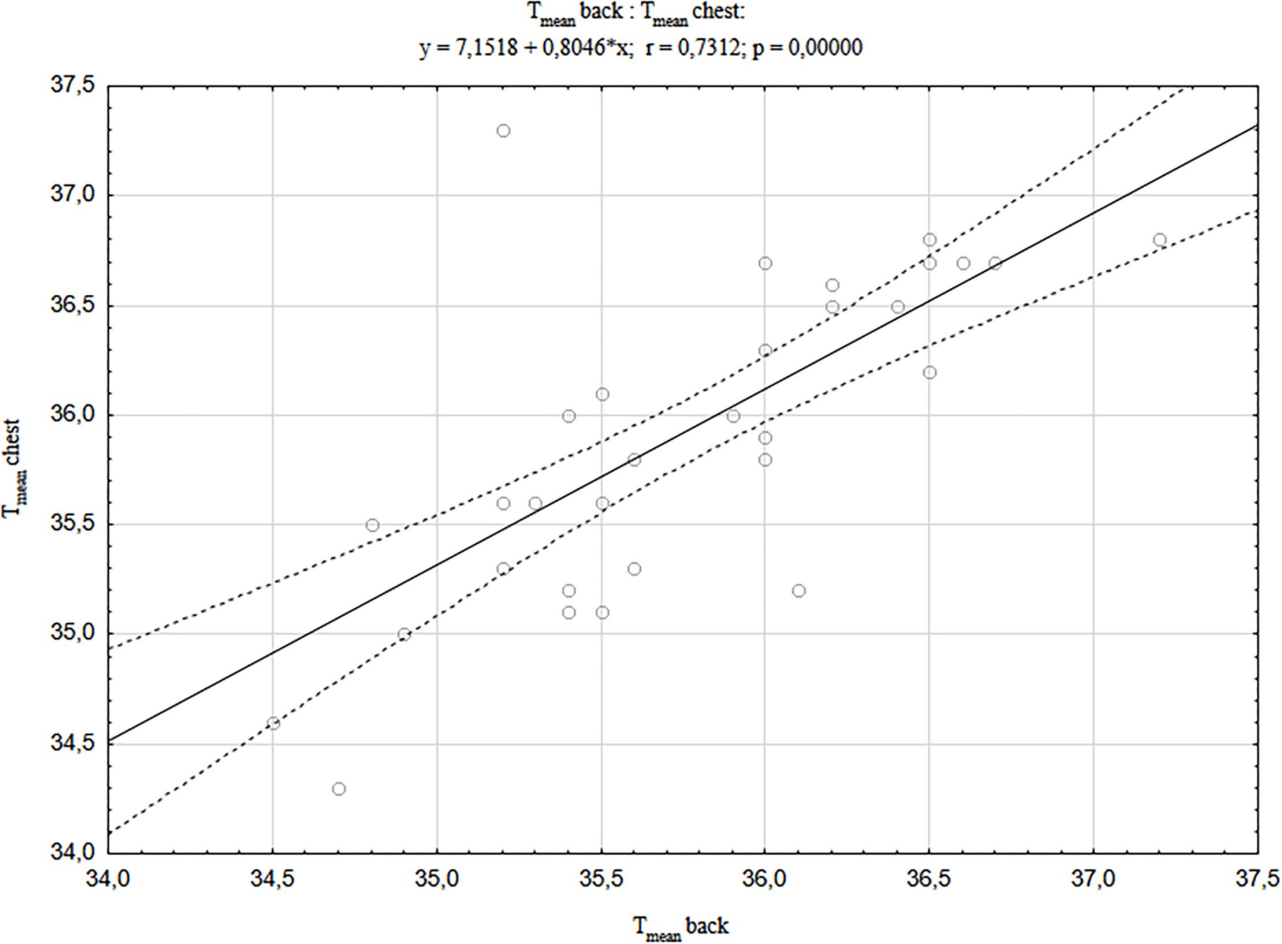
A correlation between T_mean_ back and T_mean_ chest in newborns born by CS.

In addition, it was found that the temperatures of the examined body areas (forehead, back and chest) strongly correlated positively with each other in newborns born by CS (Figs 1–3). The strongest correlations were found between the forehead and other body areas, and the values of correlation coefficient were r = 0.837 for the chest and r = 0.802 for the back (respectively at p <0.001). Similar relationships were also found in newborns born by vaginal delivery, although they were of much smaller statistical strength and the correlation values between the head area and other body areas were the weakest of all [40].

Next, in order to verify the actual effect of the mode of delivery on the examined parameters of newborns, an analysis Spaerman's rank-order correlation of the mode of delivery (vaginal vs. caesarean section) with the newborn's birth parameters was performed: the week of foetal life (pregnancy week on the day of delivery), sex, body weight and length, biochemical blood parameters and surface temperature of selected body surface areas. As results from the statistical analysis show, the correlation only occurred in the case of the value of T_mean_ forehead temperature with the mode of delivery, i.e. delivery by CS does not cause a decrease in temperature in the head area, as is the case during vaginal delivery (Table 3).

**Table 3:** Spearman’s rank-order correlation of the mode of delivery (vaginal delivery vs. cesarean section) with selected newborn parameters.

Newborn parameters	Spearman’s rank-order correlation coefficient	P
pH	-0.015	0.628
pCO2 [mmHg]	0.088	0.439
pO2 [mmHg]	0.015	0.945
HCO3 [mmol/l]	0.174	0.59
ctCO2 [mmol]	0.009	0.697
sO2%	-0.001	0.851
T_mean_ back (°C)	0.160	0.131
T_mean_ chest (°C)	0.161	0.103
T_mean_ forehead (°C)	0.362*	0.000188*

* significance level of p < 0.05.

An important parameter differentiating the course of natural childbirth is the duration of its second stage. The average duration of this stage in the examined women was 29.7 ± 12.08 minutes and had a statistically significant effect on the surface temperatures of two body areas, i.e.: T_mean_ chest (r = -0.256; p = 0.041) and in particular T_mean_ forehead (r = - 0.434; p = 0.00023). It was clearly demonstrated that the longer the second stage of labour, the lower the temperature values of these body areas (Table 4). At the same time, a significant correlation was found between the duration of the second stage of natural childbirth and pO2 in the newborn blood (r = - 0.406; p = 0.0016).

**Table 4:** Correlation of the duration of the second stage of labour with biochemical parameters and temperatures of selected body surface areas in newborns born by vaginal delivery.

Newborn parameters	R (correlation coefficient)	P
pH	-0.257	0.136
pCO2 [mmHg]	0.112	0.694
pO2 [mmHg]	-0.406	0.016**
HCO3 [mmol/l]	-0.229	0.185
ctCO2 [mmol]	0.017	0.924
sO2%	-0.311	0.69
T_mean_ back (°C)	-0.199	0.115
T_mean_ chest (°C)	-0.256	0.041**
T_mean_ forehead (°C)	-0.434	0.00023***

** significance level of p < 0.01

***significance level of p<0.001.

## Discussion

One of the most important factors determining the health and survival of a newborn, apart from the heart rate, breathing rate and blood pressure, is to maintain proper body temperature. Both hypo- and hyperthermia during the first days of life are potentially dangerous for newborn health and life. Despite, as mentioned in the introduction, numerous studies assessing the effect of the mode of delivery on vital signs, health status of newborns as well as the long-term health effects, there are no studies assessing the thermal response to delivery, including its course, and comparative studies for its type (VD vs. CS). Research from recent years conducted in the area of the thermal characteristics of newborns and infants mainly focus on monitoring body temperature, including its surface, in premature and very low birth weight (VLBW) newborns in the early neonatal period [42–45]. However, studies from earlier years describing the skin temperature values of different body areas in neonates, due to the different range of sensitivity of the tools used, had a large impact on variation in results [26, 44].

It is postulated that monitoring of central and peripheral temperature in infants is essential [46] and may prevent morbid outcomes due to early detection of ischaemia and possibly sepsis [47]. Therefore, in the light of indications recommended by WHO (maintenance of appropriate body temperature as a primary principle of newborn care and thermal protection for all infants), it seems reasonable and important to undertake this research subject by us [48].

Foetal temperature is approximately 0.3–0.5° C higher than in an adult [26, 49], i.e. so much higher than maternal temperature on average. After delivery the core and skin temperatures of a term neonate can decrease at a rate of approximately 0.1°C and 0.3°C per minute, respectively; however, it should be kept in mind that, according to WHO (1997), a decrease of core body temperature to 36–36.4°C is defined as mild and to 35.9–32°C as moderate hypothermia [50, 51]. The American Academy of Pediatrics defined the lower limits of normal temperature for an infant as 36.4°C [52]. According to the literature data, general temperature ranges for infants should be as follows: rectal temperature 36.5–37.5°C, skin temperature 36.2–37.2°C, and temperature of axillary sites 36.5–37.3°C [39].

The temperatures from selected areas of the body recorded by us were within a fairly wide range, often going beyond those indicated as normative for skin temperature, from the lowest values around 33.2°C in the forehead region for newborns born by vaginal delivery and 34.2°C in newborns born by caesarean section, up to the highest values in the chest region, amounting to 37.2 and 37.3°C, respectively. Our research confirms previous reports that immediately after birth, body temperature can drop between 1°C and 3°C and the greatest drop occurs in the first few minutes after birth but can last for several hours [29]. The highest drop in body surface temperature of newborns was recorded in the head area: 2.45°C (VD) and 1.89°C (CS). For the remaining analysed areas, the average drop in temperature was for the back: 1.98°C (VD) and 1.77°C (CS), and the lowest for the chest: 1.86°C (VD) and 1.64°C (CS).

These results confirmed earlier observations that the chest is the warmest and the forehead the coldest of the selected regions. The head accounts for 21% of the total surface area of the infant’s body and large proportion of the total body heat loss will be from this region, allowing a quick response following thermal changes. Attention should be paid to the studies of other scholars who, although they did not show significant differences in temperature within the leg, back, arm, head and abdomen, using infrared thermography for detailed registration of thermoregulation in premature infants, found, like us, that the area remaining relatively coldest during the first incubator phases and standardised skin-to-skin care was the supranasal region of the head. These results suggest that an active regulation process via skin temperature differences exist in the head region [53].

It has been shown that regardless of the mode of delivery, there are strong, positive correlations between the temperatures of selected newborn’s body areas (forehead, back and chest), immediately after delivery, and their strength and significance level is significantly higher in the case of delivery by CS compared to the newborn’s VD described above [40]. The previous study has also shown that following the vaginal delivery, the temperature of the newborn’s head significantly decreases compared to the rest of the body surface, and in the case of caesarean section no such thermal changes were observed in newborns. The statistically significant differences between the values of forehead temperature in newborns born by CS vs. VD obtained in the described study prompted us to look for a factor that may have an impact on lowering the temperature of this area in newborns born by vaginal delivery. It was assumed that this could be the duration of the second stage of labour and the correlation between the duration of the second stage of labour and the value of all T_mean_ temperatures of selected body surface areas was calculated, and additionally the newborn parameters. It was shown that the temperature of the forehead area decreased the more the longer the second stage of labour lasted. However, it should be noted that the duration of the second stage of labour in the examined women ranged from 9 to 52 minutes, which is within the normative values considered normal. The extended second stage of labour is defined as lasting more than 2 hours. Prolongation of the second stage of labour promotes intrauterine hypoxia [54]. The presented studies also showed a significant negative correlation between the duration of the second stage of labour and partial pressure of oxygen (pO2). Oxygen deficiency in the organism induces an increase in anaerobic metabolism, as well as an increase in lactic acid and a decrease in blood pH production. It should be noted, however, that despite these relationships, no significant differences in the values of blood gas parameters between groups of newborns were found depending on the mode of delivery.

Every healthy, full-term newborn is inevitably exposed to physiological stress related to birth, as well as thermal stress, resulting from changes in the environment and the temperature difference between body and ambient temperatures, which is confirmed by our research.

Immediately after delivery, newborns begin to lose heat, through urine and stool (3% of heat loss), high transepidermal water loss and evaporation (27% of heat loss), convection, conduction, and radiation (70%), dependent on the ambient air pressure, temperature and humidity, the temperature of surrounding surfaces and are in the group of patients at increased risk of developing hypothermia mainly in the first 24 hours of life [33]. Under optimal environmental conditions, the heat loss from term neonates is about 35 W/m^2^. Losses of 70 W/m^2^ are close to the maximum for which a neonate can compensate. In response to birth, during every one minute, under normal delivery room conditions, a newborn loses approximately 200 cal/kg [55, 56] and must rapidly elevate its heat production. Changes in temperature within the body are detected by specialised thermoreceptors located throughout the skin and the body core, including the viscera, brain, and spinal cord [57]. Localised heating or cooling of any of these structures induces global feed-back responses that oppose the applied temperature change. When the infant’s surface temperature decreases in response to sudden exposure to extrauterine environments, signals from peripheral and central thermoreceptors reach the hypothalamus through afferent pathways.

Except behavioral effectors, three physiological ones are particularly important for thermoregulation in humans under cold conditions: control of skin blood flow (through peripheral vascular constriction), involuntary muscle movements (shivering) and BAT thermogenesis. The engagement of specific thermoregulatory mechanisms is hierarchical, meaning that different effectors become activated at different temperature thresholds. In adults, exposure to cold activates vasoconstriction before shivering or BAT thermogenesis, in concurrence with the relative energy cost of these different mechanisms [58]. While shivering thermogenesis does occur in neonates, it is usually a response to extreme thermal stress and is insufficient to protect the infant due to the relative immaturity of the skeletal muscles resulting in diminished heat production. Similarly, vasomotor responses, controlled primarily by the release of norepinephrine from sympathetic fibres innervating vascular smooth muscle in the skin, which promotes vasoconstriction, are not effective in neonatal. The significant role of non-shivering thermogenesis (NST) at birth has been well recognised. It is the primary mode of heat production requiring an increase in norepinephrine and thyroid-stimulating hormone. Brown fat stores laid down from week 25 week of gestation [59, 60] constitute only 1.4% of the body mass of human newborns, over 2,000 grams, and after delivery diminish quickly during cold stress [61]. BAT differs morphologically and metabolically from white adipose tissue, it contains numerous fat vacuoles, triglycerides and the presence of abundant sympathetic innervations and blood supply. A signal transmitted via the sympathetic nervous system to brown adipocytes causes norepinephrine to be released, which initiates enhancement of lipolysis in brown adipocytes, mainly by β-adrenergic receptor, and triggers non-shivering thermogenesis, which is the main homeothermic heat production mechanism in newborns [62]. Heat production occurs through uncoupling ATP synthesis via the oxidation of fatty acids in the mitochondria, utilising the uncoupling protein (UCP). The resulting norepinephrine release causes vasoconstriction, glycolysis, and uncoupling of mitochondrial oxidation in brown adipose tissue and lead to generating heat production. Effectiveness of this mechanism is possible in full-term newborns, because the appropriate amount of brown fat and the levels of 5’/3’-monodeiodinase and thermogenin build up only later in foetal development [26, 63, 64].

Catecholamines released by the adrenal medulla during birth play a key role in the adaptation of a newborn to extrauterine life. Respiratory, metabolic and cardiovascular adaptations to hypoxia and other stresses associated with delivery are dependent upon a profound surge of adrenomedullary activity which occurs despite the immaturity of connections between the central nervous system and the adrenal gland. The "non-neurogenic" response seen in the foetus and neonate is thus essential to survival, and any interference either with catecholamine release or with catecholamine actions at adrenergic targets results in loss of the ability to survive hypoxia or other stressors. The immature secretory mechanism disappears as a result of development of neural connections, and factors which accelerate ontogeny of neural competence thus lead to premature loss of non-neurogenic secretory capabilities and a consequent increase in vulnerability. The foetus and neonate also have unusual proportions of adrenergic receptor subtypes in many tissues; these confer reactivity to specific stimuli associated with birth and with periods in which tissue differentiation may be under adrenergic control. Sympathetically mediated cutaneous vasoconstriction represents the “first line of defence” during exposure to cold environmental temperatures. Decreases in mean skin and/or internal temperatures cause reflex activation of sympathetic vasoconstrictor nerves, resulting in cutaneous vasoconstriction and decreases in skin blood flow [65–67].

Scientific research indicates that increased levels of catecholamines in neonates born by vaginal delivery reflect not only the response to acute stress, but also the body's attempt to increase the chances of survival after birth, and neonates born by caesarean section may be in a disadvantageous adaptation situation [68]. The study by Hägnevik et al. suggests that vaginally delivered infants showed high levels of catecholamines, arterial glucose and free fatty acids and glycerol at birth compared to infants born by caesarean section under epidural or general anaesthesia [17, 69]. The stress of journeying through the birth canal is not harmful to most infants. In fact, the surge of “stress” hormones it triggers can be important to the neonate's survival outside the womb. The thermogenic response begins within minutes of birth and continues for many hours causing a two- to three-fold increase in oxygen consumption during cold stress at birth.

Due to limitations resulting from the thermoregulatory abilities, newborns are exposed to chilling immediately after birth. Pre-term neonates and those with z VLBW require special thermal supervision due to insufficient brown fat stores which can lead to poor heat production, increased surface area to body mass ratio which can also lead to heat loss, inability to change posture due to the immaturity of musculoskeletal system, and immature skin that is poorly keratinised leading to large heat and moisture loss [63, 70]. It should be remember that both the term and pre-term neonate may be incapable of thermoregulation [70, 71].

It can be stated that in the case of birth by caesarean section, sudden exposure of the whole body to a new environment creates conditions for multiplied surface effects of temperature and humidity of the environment on the exteroreceptors of newborn’s skin compared to the progressive birth by vaginal delivery, which seems to be unfavourable from the point of view of the newborn's ability to acclimate to new ambient conditions. Previous clinical experience suggests that infants delivered by caesarean section have difficulties maintaining normal body temperature during the first 90 min after birth. The study by Christensson et al. (1993) indicates that in the subsequent minutes after delivery axillary and skin temperatures are significantly higher in the vaginally delivered neonates than in those delivered by caesarean section [72].

Neonates who had no skin-to-skin contact with their mother immediately after delivery are 4.3 times more likely to be hypothermic when compared to those who have this contact. The possible reason could be the in utero body temperature of the foetus being consistent with maternal temperature. Neonates who had skin-to-skin contact immediately after delivery with their mother gain heat through conduction which is consistent with their temperature in the womb during exposure of the newborn to extrauterine environment [73].

## Conclusion

Based on the results of this study, it can be stated that despite the same thermal / humidity conditions prevailing in the delivery room, the drop in body surface temperature of newborns immediately after delivery is within a fairly wide range. Individually differentiated from a maximum of 4.3°C to a minimum of 0.2°C, being 1,64–2.45°C on average, regardless of the mode of delivery.

Immediately after vaginal delivery, there is a decrease in the temperature of the forehead area in newborns, which is not recorded in the case of caesarean section. Particularly important in the case of VD, in terms of its impact on the temperature range, is the duration of the second stage of labour, the prolongation of which leads to a decrease in the temperature of the neonate’s forehead skin, in relation to the chest and back areas. In the case of CS, immediately after delivery, no thermal differentiation is observed in body surface areas under discussion.

In the case of healthy neonates, with normal birth weight and full-term, vaginal delivery creates more favourable conditions stimulating the mechanisms of adaptation for a newborn than caesarean section. Since it is known that mammalian newborns increase heat production within minutes after birth, some heat loss after delivery, rather slow and progressive (VD) than simultaneous for the whole body surface (CS), may be an important stimulus for metabolic adaptation; in addition, its extent is perhaps more suitable for immediate postnatal physiological adaptation of newborns to avoid a drop in body temperature proven to be detrimental to infants [74].

The temperatures of selected body surface areas correlate highly positively, regardless of the mode of delivery. It is necessary to monitor the temperature of selected areas of the newborn's body in the event that the second stage of labour is prolonged. To assess a newborn's temperature, it is important to use selected areas of the body's surface that are important in terms of thermoregulation, and to use a reliable, accurate measuring instrument, which is an IR camera.
